# Data on nitridation effect of AlTiTaZrHf(-N) high entropy films by X-ray photoelectron spectroscopy

**DOI:** 10.1016/j.dib.2022.108241

**Published:** 2022-05-06

**Authors:** Mohamed El Garah, Djallel Eddine Touaibia, Sofiane Achache, Alexandre Michau, Elizaveta Sviridova, Pavel S. Postnikov, Mohamed M. Chehimi, Frederic Schuster, Frederic Sanchette

**Affiliations:** a LASMIS, Pôle Technologique de Sud – Champagne, Antenne de Nogent – 52, Nogent 52800, France; b Nogent International Center for CVD Innovation (NICCI), LRC CEA-LASMIS, Pôle Technologique de Sud – Champagne, Nogent 52800 , France; c Research School of Chemistry and Applied Biomedical Sciences, Tomsk Polytechnic University, Tomsk 634050, Russia; d Commissariat à l'Energie Atomique et aux Énergies Alternatives (CEA) Saclay, Gif-sur Yvette 91191, France; e ITODYS, CNRS, UMR 7086, Université de Paris,15 rue JA de Baïf, Paris 75013, France

**Keywords:** Thin films, High entropy nitrides, Magnetron sputtering, Surface analysis

## Abstract

The data presented in this article are related to the published research of “Effect of nitrogen content on structural and mechanical properties of AlTiZrTaHf(-N) high entropy films deposited by reactive magnetron sputtering”. This database contains X-ray photoelectron spectroscopy (XPS) measurements, performed in order to determine the extents of nitrides formed in AlTiTaZrHf high entropy films. The latter were prepared by DC magnetron sputtering technique in reactive mode by adding the nitrogen to argon gas. The nitrogen flow rate is calculated by R_N2_ = N_2_/(N_2_+Ar). XPS measurements were done one month later. Oxides were detected on the top surface of the samples. 2p, 3d and 4f core level peaks were fitted in order to determine accurately the chemical composition of the nitride films. Al2p, Ti2p, Zr3d, Ta4f, and Hf4f reveal the formation of nitrides of all elements constituting the films. Atomic percentage of each element was calculated revealing an increase of nitrogen loading and decrease of the metallic fractions of the elements as R_N2_ grows from 5% to 50%. Nitridation behaviour of each element, as a function of the nitrogen flow rate, is investigated and presented.

## Specifications Table

**Table:** 

Subject	Materials Sciences
Specific subject area	XPS measurements describing the effect of nitrogen content from 0% to 50% on the surface chemical composition of AlTiTaZrHf high entropy film.
Type of data	Table Chart Graph Figure
How the data were acquired	Measurements carried out using XPS instrument fitted with monochromated X-ray source. Data analysed with Avantage software and prepared by using Igor Pro.
Data format	Additional analyzed data corresponding to fitted XPS peaks are available at the following link: https://figshare.com/s/a4d038f514016b19bd6b
Description of data collection	X-ray photoelectron spectroscopy (XPS) measurements were carried out by using NEXSA apparatus (Thermo, East Grinsted, UK) fitted with a monochromatic Al Kα source (hν = 1486.6 eV). These experiments were done at TPU in Tomsk, Russian Federation. The measurements were conducted at a pressure of 10^−9^ mbar. The samples were unpacked and handled with clean tweezers; placed and clipped to the sample holder using copper clips. The sample holder supporting up to 6 samples was placed in the fast entry lock and outgassed overnight. The sample holder was then shifted to the analysis chamber. The X-ray gun was switched on, and for each sample the optimal z position was determined automatically using an "auto-height" routine. We systematically checked whether the flood gun was necessary to conduct the analyses. For all samples, it was not necessary to do it, and analyses could be performed without the use of the flood gun. The samples were analysed as prepared, without any further cleaning procedure. The number of scans was between 4 and 16 depending on the relative peak intensity of the core level peaks. The survey and narrow scans were recorded under these conditions: pass energy/step size = 200/1 eV, and 40/0.1 eV, respectively. All spectra were checked, visually, for the high signal-to-noise (S/N) ratio, at the end of each analysis run before removing the samples from the analysis chamber. For the peak-fitting, we used the constraints as follows: Shirley baseline, ±0.1 eV for binding energy position, ±0.1 eV for FWHM, and the Lorentzian to Gaussian (L/G) peak shape ratio was adjusted (in the 0–30% range). For the spin-orbit doublets Hf4f, Ta4f, Zr3d and Ti2p, we have considered the binding energy splitting as well as the theoretical peak area ratios expected for f, d, and p orbitals (4/3 for 4f_7/2_/4f_5/2_; 3/2 for 3d_5/2_/3d_3/2_; 2/1 for 2p_3/2_/2p_1/_2). Taking into consideration the step size (0.1 eV) and setting the peak position to ± 0.1 eV in the course of the peak fitting, the accuracy could be estimated to ±0.2 eV.
	XPS data were processed without smoothing and without any static charge calibration, because the materials were electrically conductive. No C1s peak position was used for calibration. Note however that in the absence of any binding energy scale correction, the C1s peak from adventitious hydrocarbon contamination was found to be naturally centred at 284.8 eV [1].
Data source location	• Institution: LASMIS, Université de Technologie de Troyes, Antenne de Nogent – 52, Pôle Technologique de Sud – Champagne, 52800 Nogent, France. • City/Region: Nogent/Grand Est • Country: France
Data accessibility	Repository name: Figshare server. Data identification number: 10.4121/19196615 Publisher: 4TU.ResearchData Direct URL to data: https://figshare.com/s/a4d038f514016b19bd6b
DOI Related research article	M. El Garah, D.E. Touaibia, S. Achache, A. Michau, E. Sviridova, P.S. Postnikov, M.M. Chehimi, F. Schuster, F. Sanchette, Effect of nitrogen content on structural and mechanical properties of AlTiZrTaHf(-N) high entropy films deposited by reactive magnetron sputtering, Surf. Coat. Technol. 432 (2022), 128051. DOI: 10.1016/j.surfcoat.2021.128051.

## Value of the Data

• The data provide a deep understanding of the formation of nitrides of different elements constituting the high entropy films.
• The data give a detailed analysis on nitrogen-element bonds as nitrogen loading increases.
• It is useful to understand how nitriding occurs in the case of high entropy alloys
• The data can be exploited to understand the nitriding effect of nitride-forming metal in high entropy materials field.
• These data can be highlighted with other microstructural investigations for high entropy alloys

## 1 Data Description

This article presents the data associated to published work on effect of nitrogen content structural and mechanical properties of AlTiZrTaHf(-N) high entropy films deposited by reactive magnetron sputtering [2]. The films are deposited on silicon wafers by using DC magnetron sputtering of 99.99% pure high entropy equiatomic AlTiTaZrHf alloy target. Deph4 (DEPHIS, Etupes, France) reactor equipped with 4 circular cathodes 200 mm in diameter has been used to prepare the films in reactive mode by changing the ratio of argon-to-nitrogen gas mixture. For more details, the preparation and investigations of all samples are presented in the published work [2].

Additional amazed data which correspond to the fitting of the peaks of all elements of XPS measurement are presented figshare sever. All these data are uploaded with excel files including then the fitting peaks of N1s, Al2p, Ti2p, Zr3d, Ta4h and Hf4f as a function of the nitrogen flow rate (R_N2_ varies from 5 to 50%). A table of all parameters (peak position, FWHM, intensity, etc.) of each fitting for each peak is provided. The data are accessible with the following link: https://figshare.com/s/a4d038f514016b19bd6b.

## 2 Experimental Design, Materials and Methods

**Fig. 1:**
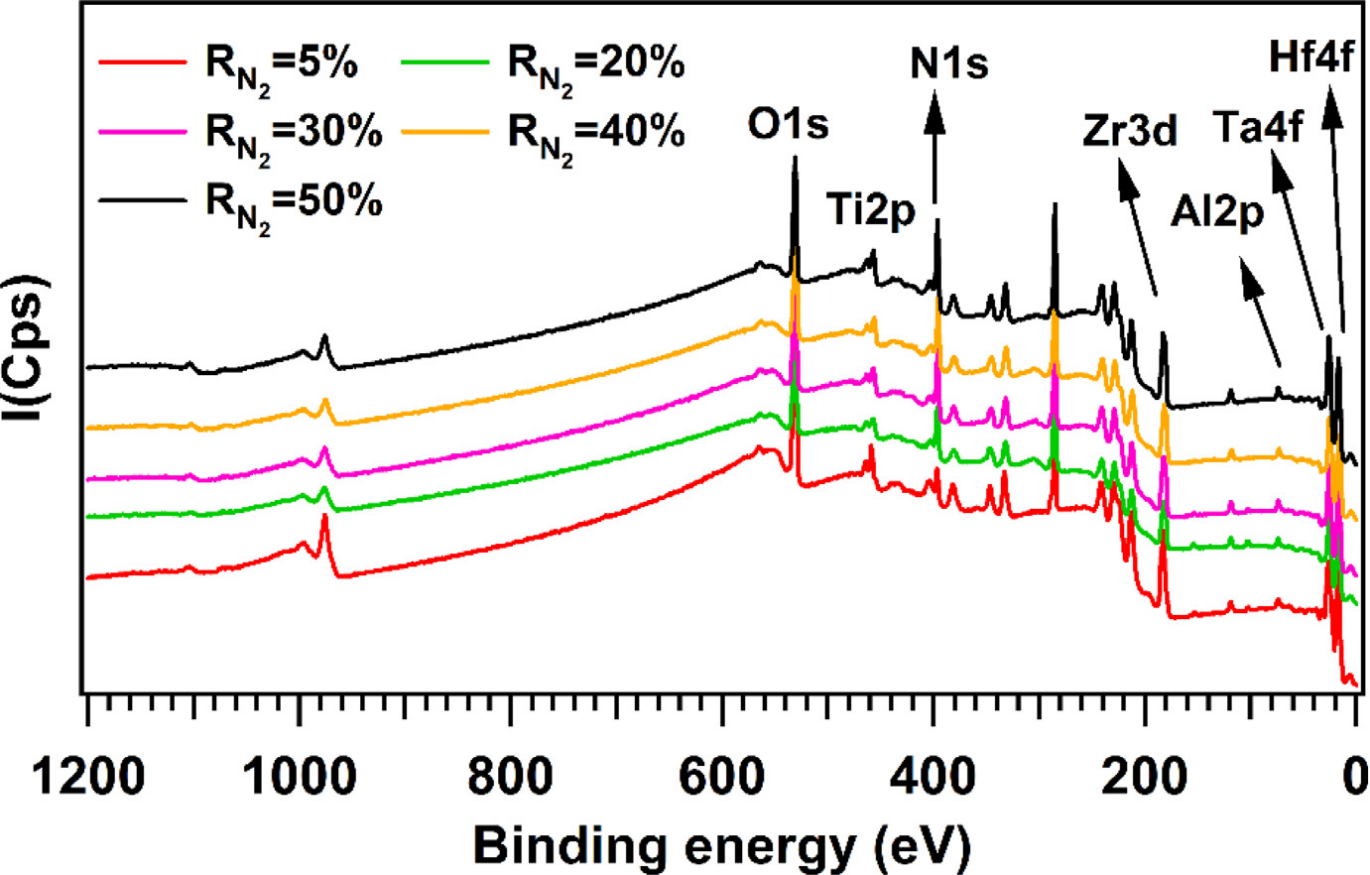
XPS survey spectra of AlTiTaZrHf(-N) as a function of R_N2_.

XPS is a powerful tool to provide information on the composition and the relevant chemical bonds between the different elements in the top surface of materials. Fig. 1 presents survey XPS spectra of all films from R_N2_ = 5% to 50%. All elements of the present high entropy alloys are detected; namely, Hf, Ta, Al, Ti, and Zr. Nitrogen and oxygen signals are also found on the survey. The binding energy positions are compared to published articles and to XPS NIST data [3].

**Fig. 2:**
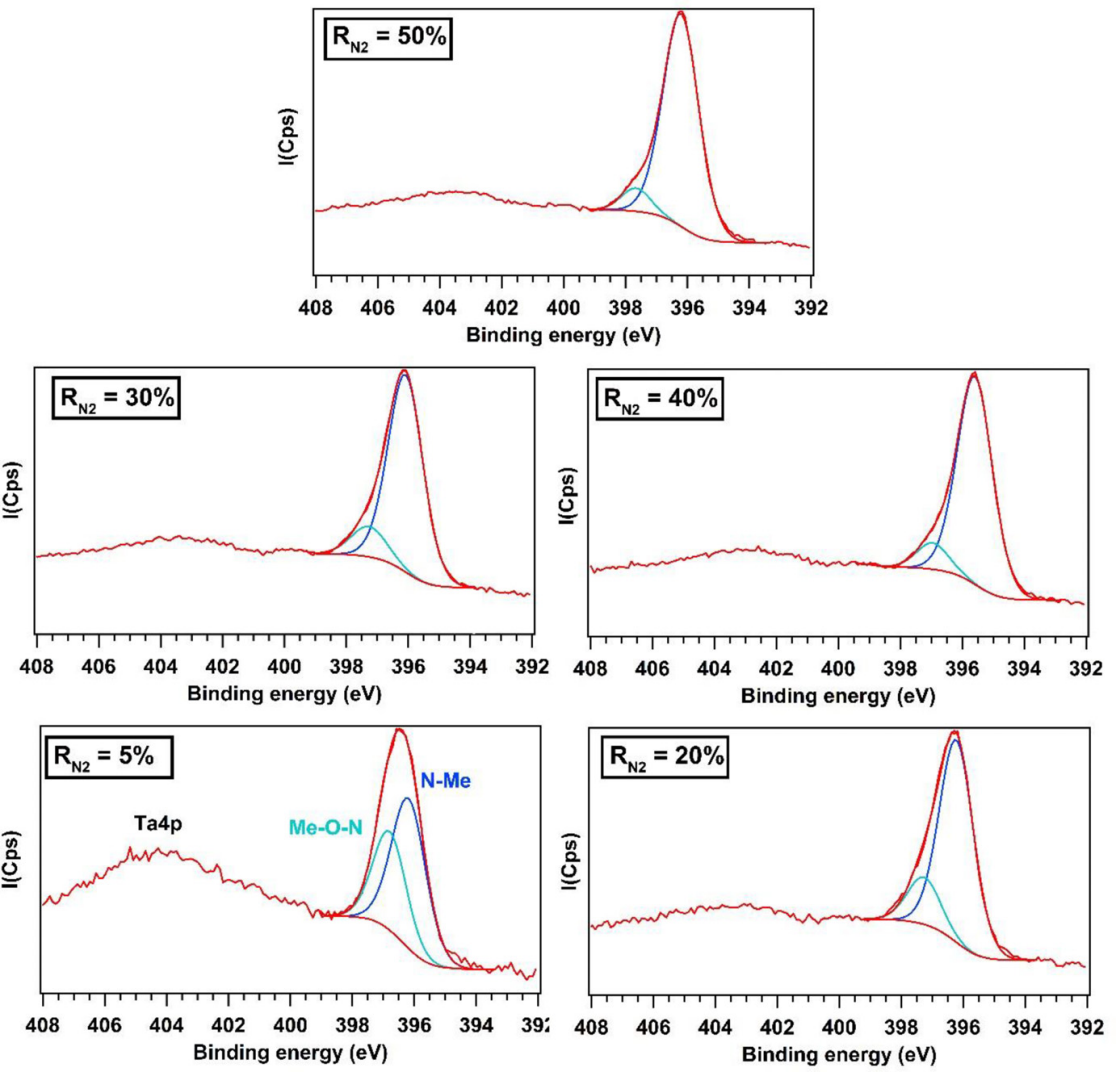
N1s XPS spectra of AlTiTaZrHf(-N) as a function of R_N2_.

High resolution XPS N1s spectra are presented in Fig. 2. Fitting of high-resolution spectra of principal elements of the different films were caried out and the resulting binding energy positions compared to those reported in the literature. A large peak is found around 405 eV that is attributed to Ta4p [4]. From R_N2_ = 5% to 50%, the spectrum can be fitted into two peaks at 396.2 eV and 397.1 eV presenting the binding energy of nitrogen in a metal nitride state and oxide, respectively [2]. As the nitrogen flow R_N2_ increases, the first peak increases while the second one decreases (Fig. 2). More nitrides are formed according to the increase of the nitrogen flow rate in the gas mixture.

**Fig. 3:**
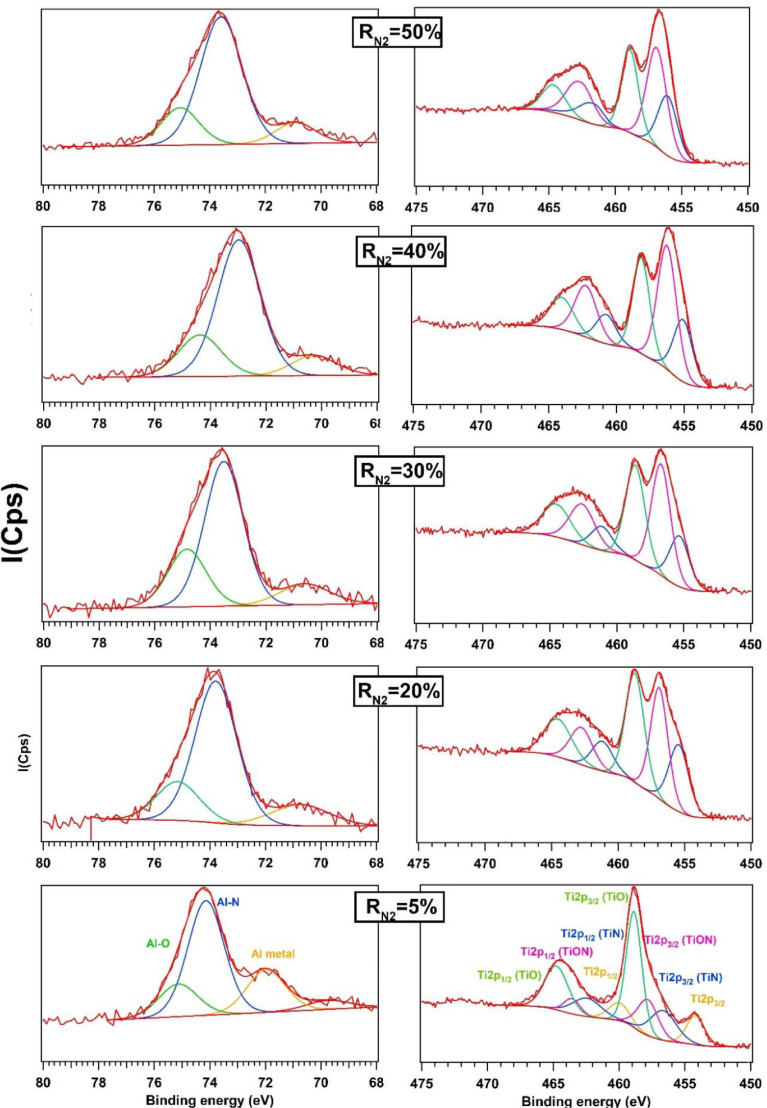
Al2p and Ti2p XPS spectra of AlTiTaZrHf(-N) as a function of R_N2_.

Fig. 3 shows the peaks of Al2p and Ti2p as function of R_N2_. The spectra of Al2p are fitted with three peaks. One at 71.9 eV attributed to the metal [5] and two others correspond to Al-N and Al-O between 74.2 eV and 75.2 eV, respectively [6]. As the nitrogen flow rate increases from 5 to 50%, the peak of Al metal shows a small shift to the lower binding energy. In these conditions, the peak area of Al-N increases, as can be seen on the spectra in the Fig. 3. Ti2p XPS is composed by four spin-orbit doublets when R_N2_ = 5%. The Ti metal peaks are centred at 454.2 ev (Ti2p_3/2_) and 460.0 eV (Ti2p_1/2_), respectively. The nitride TiN is formed when the nitrogen is introduced. The components of Ti-N are found at 456.6 eV (Ti-N(Ti2p_3/2_)) and 462.4 eV (Ti-N(Ti2p_3/2_)). Intermediate states were also found and correspond to Ti-O-N, its components are at 457.8 eV (Ti-O-N(Ti2p_3/2_)) and 463.5 eV (Ti-O-N(Ti2p_1/2_)) [7]. As R_N2_ increases from R_N2_ = 20% to 50%, Ti is totally transformed to nitride and no metallic peaks is observed (Fig. 3). Besides, the peaks area of nitride (Ti-N) increases as the nitrogen ratio increases which means its content grows into the films.

**Fig. 4:**
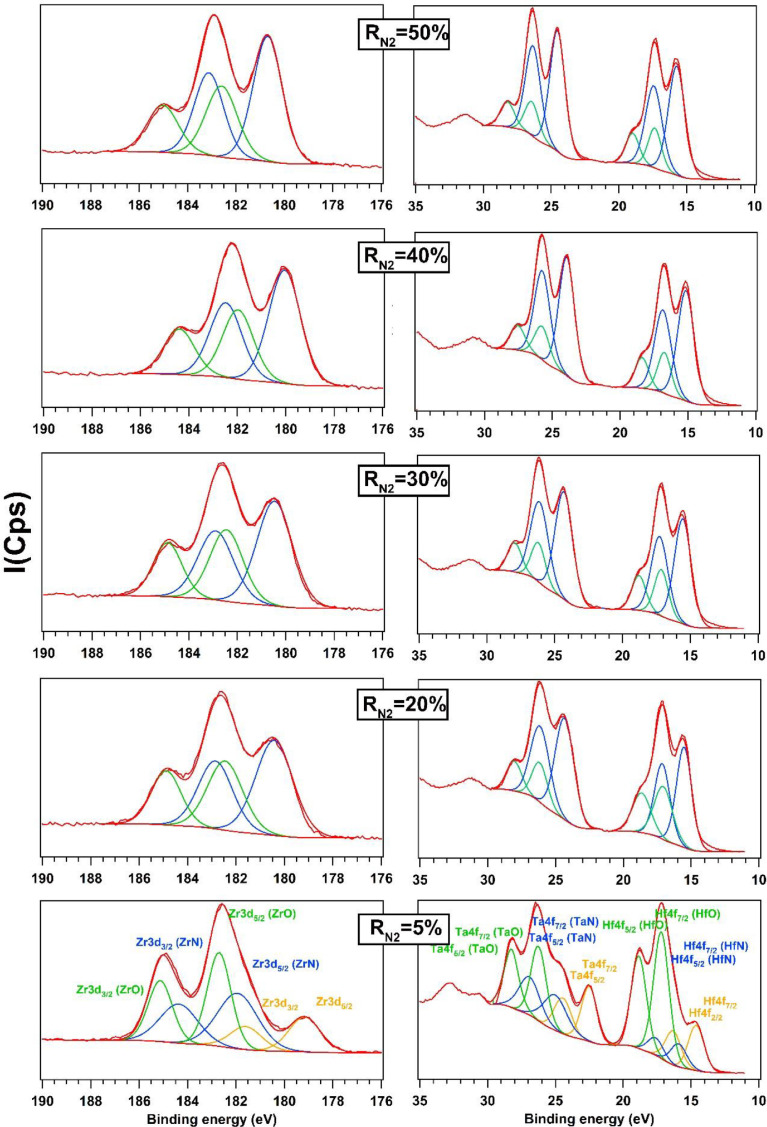
Zr3d, Ta4f and Hf4f XPS spectra of AlTiTaZrHf(-N) as a function of R_N2_.

Zr3d was fitted with three spin-orbit doublets at R_N2_ = 5% and two spin-orbit doublets when R_N2_ increases from 20 to 50%. Zr metal peaks are found at 179.1 eV and 18.6 eV for Zr3d_5/2_ and Zr3d_3/2_ respectively. Zr metal state is observed in the film at R_N2_ = 5% and is characterized by two peaks at 179.1 eV and 181.6 eV for Zr3d_5/2_ and Zr3d_3/2_ respectively. After increasing the nitrogen, the metal components disappear, and nitrides are formed. Zr-N are presented by two peaks at 181.9 eV for orbital 3d_5/2_ and 184.3 eV for orbital 3d_3/2_ [8]. When R_N2_ increases from 20% to 50%, the quantity of Zr-N increases in the films (Fig. 4).

Ta4f and Hf4f XPS spectra are plotted together and presented in the Fig. 4 (right side). At R_N2_ = 5%, the spectra of each element are fitted by three spin-orbit doublets. Ta metal components are found at 22.5 eV for orbital 4f_7/2_ and 24.4 eV for orbital 4f_5/2_ [9]. Ta-N is fitted with two peaks 4f_7/2_ at 24.9 eV and 4f_5/2_ at 26.8 eV [10]. As the nitrogen increases, a total transformation of Ta metal to nitride occurred. The peak areas of Ta-N increase when R_N2_ grows from 20 to 50%. In the case of Hf4f, the spectrum at R_N2_ = 5% was fitted into Hf metal and nitrides (oxides are presents, green color). The peaks of Hf metal are fund around 14.6 eV and 16.3 eV for orbital 4f_7/2_ and 4f_5/2_ respectively. They disappear when R_N2_ increases from 20 to 50% and Hf-N nitrides are formed. The peaks of these nitrides are located at 15.9 eV for orbital 4f_7/2_ and 17.6 eV for orbital 4f_5/2_.

**Fig. 5:**
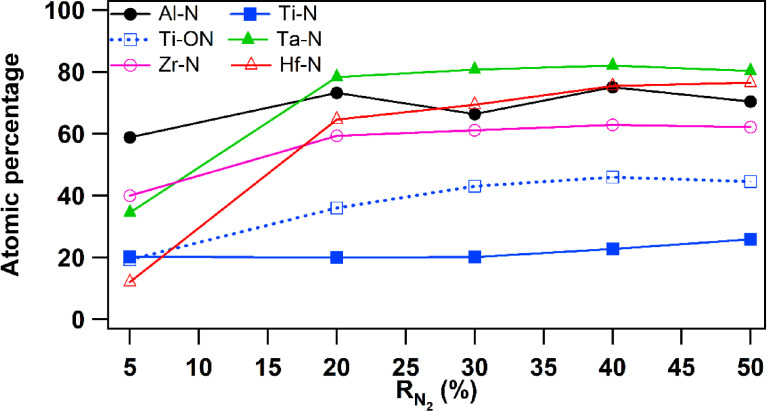
Atomic percentage of individual elements of AlTiTaZrHf(-N) films as a function of R_N2_ according to XPS analysis.

Fig. 5 presents the evolution of nitridation effect of each element as a function of R_N2_. The atomic percentage was estimated from the fitting of all XPS data according to R_N2_. Two metals Ta and Hf show a quick increasing when R_N2_ increases from 5 to 20% followed by a stable evolution as R_N2_ continuous to grow from 20 to 50%. However, Ti behaviour under nitridation effect displays a very slight or even quasi-stable evolution as R_N2_ increases. A part of Ti-N is oxidised but the quantity of oxynitride decreases when R_N2_ increases. The Al element shows a percentage varying between 58.9 and 70% and is the first nitride element compared to others.

After introducing a small amount of nitrogen (R_N2_ = 5%), Hf-N and Ti-N are found at low extents compared to the other nitrides Al-N, Ta-N and Zr-N. However, when R_N2_ increases, Ti-N and Al-N exhibit a quasi-stable evolution, whereas the quantities of other nitrides increase to reach the saturation point followed by steady state.

## Ethics Statements

The data are the authors' own original work, which have not been previously published elsewhere.

## CRediT authorship contribution statement

**Mohamed El Garah:** Conceptualization, Investigation, Formal analysis, Validation, Writing – review & editing, Writing – original draft. **Djallel Eddine Touaibia:** Writing – review & editing. **Sofiane Achache:** Writing – review & editing. **Alexandre Michau:** Conceptualization, Funding acquisition. **Elizaveta Sviridova:** Investigation, Methodology, Data curation. **Pavel S. Postnikov:** Investigation, Data curation. **Mohamed M. Chehimi:** Investigation, Formal analysis, Writing – review & editing. **Frederic Schuster:** Conceptualization, Funding acquisition. **Frederic Sanchette:** Conceptualization, Funding acquisition, Writing – review & editing.

## Declaration of Competing Interest

The authors declare that they have no known competing financial interests or personal relationships that could have appeared to influence the work reported in this paper.

## Data Availability

Raw data of XPS fitting (Original data) (4TU.ResearchData).

## References

[1] Greczynski G., Hultman L. (2017). C 1s peak of adventitious carbon aligns to the vacuum level: dire consequences for material's bonding assignment by photoelectron spectroscopy. ChemPhysChem.

[2] El Garah M., Touaibia D.E., Achache S., Michau A., Sviridova E., Postnikov P.S., Chehimi M.M., Schuster F., Sanchette F. (2022). Effect of nitrogen content on structural and mechanical properties of AlTiZrTaHf(-N) high entropy films deposited by reactive magnetron sputtering. Surf. Coat. Technol..

[3] NIST X-ray Photoelectron Spectroscopy Database, NIST Standard Reference Database Number 20, National Institute of Standards and Technology, Gaithersburg MD, 20899 (2000), doi:10.18434/T4T88K.

[4] Zaman A., Meletis E.I. (2017). Microstructure and mechanical properties of TaN thin films prepared by reactive magnetron sputtering. Coatings.

[5] Klein J.C., Hercules D.M. (1983). Surface characterization of model Urushibara catalysts. J. Catal..

[6] Khan N.A., Akhavan B., Zhou C., Zhou H., Chang L., Wang Y., Liu Y., Bilek M.M., Liu Z. (2020). High entropy nitride (HEN) thin films of AlCoCrCu0. 5FeNi deposited by reactive magnetron sputtering. Surf. Coat. Technol..

[7] Bagdasaryan A., Pshyk A., Coy L., Konarski P., Misnik M., Ivashchenko V., Kempiński M., Mediukh N., Pogrebnjak A., Beresnev V. (2018). A new type of (TiZrNbTaHf) N/MoN nanocomposite coating: microstructure and properties depending on energy of incident ions. Compos. Part B Eng..

[8] Matsuoka M., Isotani S., Sucasaire W., Kuratani N., Ogata K. (2008). X-ray photoelectron spectroscopy analysis of zirconium nitride-like films prepared on Si(100) substrates by ion beam assisted deposition. Surf. Coat. Technol..

[9] Kerrec O., Devilliers D., Groult H., Marcus P. (1998). Study of dry and electrogenerated Ta_2_O_5_ and Ta/Ta_2_O_5_/Pt structures by XPS. Mater. Sci. Eng. B.

[10] Valleti K., Subrahmanyam A., Joshi S.V., Phani A., Passacantando M., Santucci S. (2008). Studies on phase dependent mechanical properties of dc magnetron sputtered TaN thin films: evaluation of super hardness in orthorhombic Ta4N phase. J. Phys. D Appl. Phys..

